# How to improve walking, balance and social participation following stroke: a comparison of the long term effects of two walking aids--canes and an orthosis TheraTogs--on the recovery of gait following acute stroke. A study protocol for a multi-centre, single blind, randomised control trial

**DOI:** 10.1186/1471-2377-12-18

**Published:** 2012-03-30

**Authors:** Clare Maguire, Judith M Sieben, Florian Erzer, Beat Goepfert, Matthias Frank, Georg Ferber, Melissa Jehn, Arno Schmidt-Trucksäss, Robert A de Bie

**Affiliations:** 1 Bildungszentrum Gesundheit Basel-Stadt & Bern University of Applied Science, Binningerstrasse 2, 4142, Muenchenstein Basel, Switzerland; 2 Caphri research school, Maastricht, the Netherlands; 3 Department of Anatomy and Embryology, Caphri research school, Maastricht University, P.O. Box 616, 6200 MD Maastricht, the Netherlands; 4 Rehab Basel, Im Burgfelderhof 40, 4012 Basel, Switzerland; 5 Laboratory Of Biomechanics & Biocalorimetry, Biozentrum/Pharmazentrum, University of Basel, Klingelbergstrasse 50-70, 4056 Basel, Switzerland; 6 Geriatric Competence Centre, Felix Platter Spital, Burgfelderstrasse 101, 4055 Basel, Switzerland; 7 Statistik Georg Ferber GmbH, Cagliostrostrasse 14, 4125 Riehen, Switzerland; 8 Department of Sports Medicine, Institute of Exercise and Health Science, University of Basel, Birsstrasse 320 B, 4052 Basel, Switzerland; 9 Department of Epidemiology, Maastricht University, P.O. Box 616, 6200 MD Maastricht, the Netherlands

## Abstract

**Background:**

Annually, some 9000 people in Switzerland suffer a first time stroke. Of these 60% are left with moderate to severe walking disability. Evidence shows that rehabilitation techniques which emphasise activity of the hemiplegic side increase ipsilesional cortical plasticity and improve functional outcomes. Canes are commonly used in gait rehabilitation although they significantly reduce hemiplegic muscle activity. We have shown that an orthosis "TheraTogs" (a corset with elasticated strapping) significantly increases hemiplegic muscle activity during gait. The aim of the present study is to investigate the long term effects on the recovery of gait, balance and social participation of gait rehabilitation with TheraTogs compared to gait rehabilitation with a cane following first time acute stroke.

**Methods/Design:**

Multi-centre, single blind, randomised trial with 120 patients after first stroke. When subjects have reached Functional Ambulation Category 3 they will be randomly allocated into TheraTogs or cane group. TheraTogs will be applied to support hip extensor and abductor musculature according to a standardised procedure. Cane walking held at the level of the radial styloid of the sound wrist. Subjects will walk throughout the day with only the assigned walking aid. Standard therapy treatments and usual care will remain unchanged and documented. The intervention will continue for five weeks or until patients have reached Functional Ambulation category 5. Outcome measures will be assessed the day before begin of intervention, the day after completion, 3 months, 6 months and 2 years. Primary outcome: Timed "up and go" test, secondary outcomes: peak surface EMG of gluteus maximus and gluteus medius, activation patterns of hemiplegic leg musculature, temporo-spatial gait parameters, hemiplegic hip kinematics in the frontal and sagittal planes, dynamic balance, daily activity measured by accelerometry, Stroke Impact Scale. Significance levels will be 5% with 95% CI's. IntentionToTreat analyses will be performed. Descriptive statistics will be presented.

**Discussion:**

This study could have significant implications for the clinical practice of gait rehabilitation after stroke, particularly the effect and appropriate use of walking aids.

The results could be important for the development of clinical guidelines and for the socio-economic costs of post-stroke care

**Trial registration number:**

ClinicalTrials.gov NCT01366729.

## Background - presentation of the hypothesis

Stroke is the leading cause of acquired disability in adults [1]. In Switzerland approximately 9000 people a year suffer a first time stroke [2]. Of those patients who survive the acute phase, between 20 and 30% are unable to walk [3]. Those who can are often left with moderate to severe walking disability and reduced gait speeds [3,4]. The risk of falling increases--a recent study indicating that at 3 months 28% of stroke patients have fallen [5]. The resulting disability has an enormous socioeconomic impact on patients, families and health service providers [6,7].

Rehabilitation methods which improve balance and gait are crucial for the quality of life of stroke victims and to reduce the ongoing cost of long term care. Studies have shown that early rehabilitation in specialised settings e.g. stroke units results in better functional outcome than in non-specialised units [8,9]. It has been suggested that these better results are achieved partly due to enhanced staff awareness of the importance of mobility thus preventing secondary complications such as loss of cardiovascular fitness or muscle weakness. The physical environment is also likely to be adapted enabling more independent movement [10,11]. Current rehabilitation methods which aim to improve motor control during walking do not appear to deliver additional improvements [12,13]. A recent meta-analysis showed that only cardiorespiratory physical fitness training provides robust evidence for a benefit to walking ability after stroke. Repetitive task training also appeared to have some effect. Motor and neurophysiological approaches did not demonstrate a positive effect on walking recovery [13]. This suggests that underlying mechanisms responsible for the recovery of motor co-ordination and control of walking following stroke are not significantly influenced by current therapy methods. These findings are further illustrated by studies which show that long term improvements in gait function occur in the absence of improvements in muscle co-ordination patterns [14] or improved kinematic or kinetic gait profiles [15]. Buurke et al. [14] concluded that "functional gait improvements may be more related to compensatory strategies than by restitution of muscle co-ordination patterns in the affected leg." Kautz et al. [16] concluded that "There is no evidence of improved locomotor co-ordination post intervention. The increased walking and pedalling speed were achieved by a more proficient use of the same impaired pattern without EMG timing changes, likely because of increased strength and endurance post intervention." We suggest that improvements in gait function due to current rehabilitation methods are predominantly achieved through more efficient use of abnormal movement patterns. This may be a reason for the generally low level of independence and function achieved following stroke.

Spontaneous recovery which occurs within the first weeks post stroke largely defines long term functional outcome [17]. Recent studies investigating changes in the cerebral cortex following focal injury have indicated that cortical plasticity and neuronal growth that occurs early after infarct may "underlie the brain's self-repair process" [18,19]. Evidence shows that this early cortical plasticity is an important factor in the spontaneous recovery of motor control which predicts long term outcome [18-22]. We suggest that it is this process which is inadequately influenced by current rehabilitation methods. The recovery of motor skills following stroke relies on the brains ability to reorganise its neuronal control of movement [19,20]. Reorganisation can occur within and between cortical networks both within the lesioned and non-lesioned hemisphere [18,21]. Rossini et al. stated that "Reorganization phenomena following ischemic stroke observed so far, may be classified into two main groups: overactivation of areas belonging to the neural network for a specific task--or activation of unusual areas that attempt to replace the function of the damaged tissue" [18]. The overactivation of the original neural network is primarily activity in the lesioned hemisphere. Following stroke cortical activity has been recorded in both lesioned and non-lesioned hemispheres during movement [21,23]. Increasingly findings show that increased activity in the lesioned hemisphere correlates with better recovery and improved motor performance [23-27]. Conversely increased activity in the contralesional hemisphere is associated with poorer motor recovery of the hemiplegic limb [26,27]. It appears that improved motor recovery occurs when the brain is able to make use of the original neural network to control movement. When new networks are formed for example in the unaffected hemisphere, motor recovery is reduced. The authors suggest that activity in the original network represents "true" recovery. The recruitment of new networks may represent the learning of compensatory movement strategies associated with poorer functional outcome.

Findings in favour of this hypothesis have been demonstrated in both upper and lower limbs [25-27]. As post-injury behavioural experience is critical to the reassembly of adaptive networks and strongly influences cortical reorganisation [22,28] these results indicate that treatment approaches which promote increased plasticity in the lesioned hemisphere will promote greater functional recovery. This assumption is supported by a recent meta-analysis [21] which examined whether participating in stroke rehabilitation which emphasised use of the paretic upper limb was associated with increased cortical activity of the lesioned hemisphere and consequently better function. The review concluded that neural changes in the sensorimotor cortex of the lesioned hemisphere were achieved with rehabilitation interventions that emphasized use of the paretic upper limb and resulted in improved functional motor gains.

Nudo et al. [28] demonstrated with squirrel monkeys that forced retraining of skilled hand use prevented loss of hand territory representation adjacent to the infarct and in some instances the representations expanded. This functional reorganization was accompanied by recovery of skilled hand movements. Control monkeys with identical lesions who did not receive therapy lost paretic hand representation und function.

In general these results suggest that therapies which emphasise use of the hemiplegic side promote plasticity in the lesioned hemisphere through emphasising the use of previously established neural networks and are associated with improved function. It may be that therapies which promote compensatory movement or reduce activity of the hemiplegic limb inhibit plasticity in the lesioned hemisphere and promote the development of new neural networks associated with reduced function.

To date therapies which emphasise use of the hemiplegic limb have been confined to the upper extremity [29,30] and have not been applied during gait rehabilitation following stroke. Canes are very commonly used post stroke although studies have consistently shown a significant reduction in surface electromyography (EMG) activity in all muscle groups on the side contralateral to cane use in both stroke and non-stroke patients [14,31-33]. In light of the factors which increase plastic reorganisation in the lesioned hemisphere, namely interventions which emphasise use of the paretic limb, the effect of canes which reduce activity in the hemiplegic side may be to inhibit activity in the original neural networks responsible for lower limb control resulting in poorer walking function.

In gait rehabilitation, attention should also be paid to the optimal restoration of balance. In relation to balance, evidence shows that balance control does not occur automatically at spinal cord and brainstem level as has previously been thought, but rather is highly influenced by cortical activity and cognitive control [34-36]. Two main types of balance strategies are recognised--fixed support or change of support strategies [34,35]. Fixed support strategies are used when no stepping or reaching activities are needed to maintain balance. Rotatory torques are generated through muscle activity primarily around the hip and ankle. Change of support strategies are used in challenging conditions when stepping or reaching reactions are necessary to maintain equilibrium. Fixed support strategies use less cognitive resources than change in support [34,35]. Elderly people or subjects with poor balance use more change in support strategies and therefore more cognitive resources than younger, healthy subjects to maintain equilibrium under the same conditions.

The authors suggest that balance rehabilitation should attempt to restore balance strategies used by healthy individuals--namely fixed support responses requiring fewer cognitive resources in unchallenging situations. However cane use increases the base of support when walking through use of the arms. This strategy may emphasise use of cortical networks used for reaching "change of support" reactions. It emphasises use of cognitive resources on safe level ground and reduces the use of "fixed support" strategies. The authors suggest this leads to a long term reduction of automatic balance responses. It may be that because cognitive resources are needed in safe, level environments fewer additional resources are available for more difficult conditions such as walking outside or on public transport. The long term effects of canes on balance recovery and functional gait following stroke has to our knowledge been investigated in one study [37]. Balance recovery and community participation were shown to be reduced.

Taking all of these considerations together, the authors suggest that an optimal walking aid for post stroke gait rehabilitation would provide enough support to enable independent early walking without reducing hemiplegic muscle activity or inhibiting the use of balance reactions. The immediate effect of an elasticised orthotic walking aid (TheraTogs) on hemiplegic hip abductor activity has been previously investigated by the authors [38]. Activity in gluteus medius was increased by 16.5% compared to walking without aids when walking with TheraTogs (with cane use activity in gluteus medius was reduced by 22% compared to walking without aids). The increased activity with TheraTogs may be due to increased proprioceptive input provided by the orthosis or to the physical shortening of the muscle leading to increased overlap between the actin and myosin filaments and consequently a stronger contraction.

The authors hypothesize that cane use will inhibit activity on the hemiplegic side leading to reduced ipsilesional cortical activity and poorer long term functional gait outcomes. In contrast we hypothesize that TheraTogs will increase activity on the hemiplegic side during walking leading to increased ipsilesional cortical activity and improved long term functional gait outcomes.

We further hypothesize that cane use will inhibit the use of normal balance reactions leading to reduced balance recovery and poorer social participation. As no external support is provided with the TheraTogs orthosis the use of automatic balance responses will not be inhibited during walking. This will result in improved balance recovery and social participation.

The aim of this study is to investigate the long term effects of canes and TheraTogs on the recovery of motor control and co-ordination, gait, daily activity, balance and social participation when used in early gait rehabilitation following stroke.

## Methods - testing the hypothesis

### Design and setting

This study is a multi-centred single blind, randomised, control trial with parallel design.

Subjects will be recruited from the neurological rehabilitation department of 3 participating Swiss hospitals: Felix Platter Spital Basel, Kantonspital Luzern Neurorehabiltaton and Reha Clinic Zurzach. Each department is a dedicated unit specialising in the rehabilitation of post acute neurological patients. Ethical approval has been obtained from the following cantonal ethics committees: Basel (Ethikkommission Beider Basel), Luzern (Ethikkommission Kanton Luzern), Aargau (Ethikkommission Kanton Aargau).

### In- and exclusion criteria

All subjects (1) will be patients with hemiplegia following a first unilateral stroke, (2) will score at least level 3 on the Functional Ambulation Category (FAC) [39] (able to walk unaided on even ground but requiring verbal prompts and stand-by help without body contact) and (3) must have been independent walkers prior to insult without walking aids. (4) Subjects will have a Mini Mental State [40] score of 22 or above, (5) will have no orthopaedic or other neurological conditions that could limit walking ability, (6) have no gross visuospatial or visual field deficits and (7) will have no medical contraindications to walking.

### Recruitment

Potential subjects will be identified by rehabilitation staff at the participating hospitals. Suitability for participation will be checked and confirmed by the researchers. Signed, informed consent will be obtained from subjects before inclusion and randomisation. Subjects will be randomised into TheraTogs (intervention) and cane (control) group (see below). Intervention will begin when patients have reached level 3 on the FAC.

### Intervention details

Interventions will be applied for five weeks. If patients become independent walkers before this time (FAC 5) the intervention will be discontinued. If patients are discharged before this time and have not reached FAC 5 where possible the home carer will be instructed in the application of TheraTogs and the intervention will continue for 5 weeks as planned. TheraTogs will be applied as part of the washing/dressing routine in the morning by therapists or nursing staff instructed in the standard application. Subjects will walk throughout the day until preparing for bed with the prescribed walking aid. Standby assistance only will be provided during walking when necessary for safety. No other form of walking aid will be used for the duration of the study intervention. Foot or foot/ankle orthoses will remain unchanged and be worn as usual. All other forms of therapy (frequency and type) will remain unchanged and documented. Patients will receive usual care. When therapists feel that TheraTogs hinders treatment sessions the application may be removed for the duration of the session. It must be immediately reapplied with the standard application following treatment. When necessary TheraTogs may be removed for sleeping in the afternoon. It will be reapplied with the standard application on waking.

**Figure 1:**
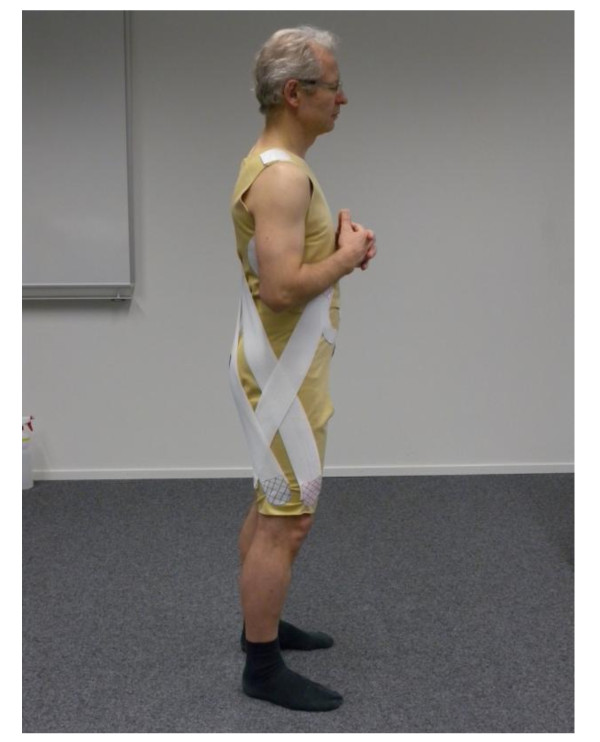
**Standard Application TheraTogs**.

TheraTogs is worn directly on the skin. Attached to the basic suit hip abductor support [38] will consist of two broad straps attached 1.to the anterior torso, pulled downwards across the abductors towards and attached to the posterior aspect of the hemiplegic leg and, 2. to the posterior aspect of the torso pulled downwards to cross the abductors and attach to the anterior aspect of the hemiplegic leg. For hip extension one wide strap will be attached to the top of the pelvic rim on the non-hemiplegic side, pulled downwards and laterally passing across the buttock and towards the anterior aspect of the hemiplegic thigh attaching laterally (Figure 1.). Standard application training sessions will be provided to all staff before study begin. Written and photographic instructions will be provided. TheraTogs suits will be marked to ensure consistent application.

In the control group cane walking will take place with cane held at the level of the radial styloid of the sound wrist. Occurrences of non-adherence to the protocol will be documented in subject notebooks.

### Measures

Outcome measures will be taken at baseline, defined as the day before intervention begin, the day after intervention is completed (max. 5 weeks), 3 months, 6 months and 2 years after intervention begin.

### Baseline descriptives

At baseline descriptive variables for each patient including age, sex area and type of infarct, side of stroke, time since stroke, height and weight will be recorded.

### Primary outcome

The primary outcome measure will be the time taken to complete the Timed "Up and Go" Test [41]. This is a basic test for functional mobility which has good reliability and validity [41,42]. The seat height will be 65% of the subjects leg length and subjects will turn towards the unaffected side [43].

### Secondary outcomes

The following secondary outcomes will be measured during walking and during sit to stand (in "Timed up and go"):

*Peak surface EMG *measurement of gluteus maximus and gluteus medius muscle (TMS International, Enschede, Holland);

*Activation patterns *of gluteus maximus, gluteus medius, vastus lateralis, semitendinosis, gastrocnemius and tibialis anterior;the peak SEMG amplitude of the maximum voluntary contraction of the unaffected gluteus maximus and medius will also be measured.

*Temperospatial gait parameters*--gait speed, cadence, step length, stride length, stance phase and swing phase of both legs; hip kinematics in the frontal and sagittal planes using twin-axis electrogoniometers (Biometrics Ltd UK, USA SG 150).

An intergrated system The Porti-system with a Polybench software package (TMS International, Enschede, Holland) will be used to simultaneously measure these parameters together with a synchronised camcorder with a sagittal view.

*Dynamic balance *will be simultaneously measured using two angular velocity sensors (fibreoptic gyroscopes) of the SwayStar balance system (Balance International Innovations GmbH, Iseltwald, Switzerland) [44,45].

*Muscle strength *of hemiplegic and non-hemiplegic hip abductors will be measured using a hand held dynamometer.

*Daily activity *during the intervention will be measured using an accelerometer (Aipermon GmbH, Germany). Activity modes and accelerometer detection accuracy have been validated [46].

*Social participation *will be measured using The Stroke Impact Scale (SIS) [47]. Validated German, French and Italian versions will be used [47,48]. The Stroke Impact Scale is a stroke specific evaluative instrument that measures the impact of stroke in multiple domains including physical, emotional, memory/thinking, communication and social participation. It is a face-to-face interviewer administered instrument that takes 15 to 20 min to conduct [47].

*"Usual care" *will be documented daily for each patient in a specifically designed questionnaire. Each 10 min treatment block will be classified into 1 of 5 categories. (See attachment "Documentation Standard Therapy.").

Technical details about the measurement of primary and secondary outcome variables are explained below.

### Testing procedure

Physical measurements take place and data collection is performed in a specific, standardised order, conform to the following protocol:

1. Hip abductor muscle strength on both sides of the body will be measured using a hand-held dynamometer. Each test will be performed three times for each leg starting with the healthy leg then alternating. Subjects will rest for 30 sec between trials. Subjects will lie in supine on an examination plinth. Two Velcro straps will be applied to stabilise the pelvis (applied over both anterior superior iliac spines and attached to the bed) and the thorax/spine (applied to the chest below the breasts and attached to the bed). Hips will be positioned in neutral extension and rotation, hip abduction in mid range and knee in extension. The dynamometer will be held perpendicular to the thigh above the lateral femoral condyl of the leg being tested with one foot of the tester against a wall for stability. Subjects will be instructed to push with maximum effort. One practice trial will be carried out prior to testing. A four second hip abduction isometric maximal contraction will be recorded. Mean maximum force over 3 measurements for each leg will be calculated [49].

2. Subjects will rest for five minutes after which a Maximun Voluntary EMG Contraction (MVC) of the non-hemiplegic gluteus maximus and gluteus medius will be measured. The skin will be prepared for surface EMG placement. Skin will be shaved over the appropriate muscles and cleaned with alcohol. Surface EMG electrodes (Kendall, Tyco/Healthcare) will be placed onto the skin overlying these muscles following the "European Recommendations for Surface Electromyography" (SENIAM) guidelines with a spacing of 20 mm [50]. The ground electrode will be placed on the clean shaven skin overlying the sacrum. EMG is measured at a sampling rate of 2048 Hz without filtering so that the signals are measured including DC. The digitalised data will be high pass filtered with a fourth order filter with a cut off frequency of 10 Hz and full wave rectified. To test gluteus maximus subjects will lie prone on an examination plinth. With hip and knee extended manual resistance will be given at the ankle joit in the opposite direction to movement. Subjects will be asked to slowly increase the force, reaching maximum effort after 3-5 sec, hold it for 3 sec and relax over 3 sec. A rest for 1 min will provided after which the test will be carried out once more. The maximum value from both tests will be used a s MVC.

To test gluteus medius, subjects will lie on the hemiplegic side. < the subject will beinstucted to lift leg sideways and manual resistance will be applied at the ankle in the opposite direction to movement. Subjects will be asked to slowly increase the force, reaching maximum effort after 3-5 sec, hold it for 3 sec and relax over 3 sec. A rest for 1 min will provided after which the test will be carried out once more. The maximum value from both tests will be used a s MVC.

3. Electrodes will be removed from the non-hemiplegic side and will be applied with the same procedure as described above to the hemiplegic muscles of gluteus maximus, gluteus medius, vastus lateralis, semitendinosis, gastrocnemius and tibialis anterior. Henna will be applied to ensure accurate replacement at subsequent sessions.

4. The electrogomiometer, foot switches and SwayStar instrumentation will also be fitted. A biaxial electrogoniometer will be placed over the anterior hemiplegic hip joint line, proximal arm in line with the anterior superior iliac spines, distal in line with the axis of the femur. Foot switches will be placed under the calcaneus and the third distal phalange of each foot to measure "initial contact" and "foot off" simultaneously with the EMG. SwayStar will be strapped to the patient's waist.

5. Patients will complete the "Timed up and go test". Time taken will be recorded, EMG activity, dynamic balance and hip kinematics will be collected and video recordings will be taken.

6. Subjects will then rest for five minutes after which they will walk for twelve gait cycles three times. Subjects will rest for five minutes between each set of twelve gait cycles. Data collected during the first two and last two gait cycles will be excluded. Data from the three sets of eight remaining gait cycles will be used to calculate mean values.

7. Patients will rest for ten minutes and then walk over a set of four low (24 cm high) barriers placed 1 m apart.

8. Measurement equipment will be removed, patients will rest for 20 min with a drink after which the SIS will be completed.

### Analysis

Hemiplegic peak EMG values of gluteus maximus and medius will be compared as a percentage of the maximum voluntary contraction peak EMG value of the same muscles of the unaffected leg.

Activation patterns of gluteus maximus, gluteus medius, vastus lateralis, semitendinosis, gastrocnemius and tibialis anterior will be assessed at baseline and at subsequent data collection points. On and off times for each muscle during each stride will be calculated. An amplitude of two standard deviations higher that the resting (reference) amplitude will be considered "on" activity. All detected on- and off- times will be normalised in time using the stride time from the related heel strike measured by a foot switch. SEMG will be rectified and filtered with a high pass fourth order filter with a cut off of 10 Hz and plotted with the timing information along the x-axis. Total burst duration (gait cycle time minus off time) and median on and off times in percentage of the gait cycle will be calculated for each subject for each muscle. Activation patterns will be compared to established normal patterns.

For kinematic measures mean hip range of movement during 1 gait cycle in the frontal plane (ab/aduction) and in the sagittal plane (flex/ext) will be measured for the intervention groups and compared with normal values for matched controls.

Balance control will be assessed with four measurements consisting of trunk pitch (forwards-backwards) angular displacement and velocity and roll (side to side) angular displacement and velocity. Measurements will be taken during all tasks. The fibreoptic gyroscopes (SwayStar) will be attached via a belt to the subjects so that the sensors are at the level L2/3. The sensors will be attached to a computer via a Bluetooth communication, which will sample the velocity signals every 100 ms and numerically integrate the velocity signals to yield angular displacement. The mean will be taken from the maximum values of the two angular displacement measures and the two angular velocities for the cane and TheraTogs intervention groups. The Sway Star has been has been used to assess static and dynamic balance in healthy individuals of differing ages [44,51] and for institutionalised older individuals [45]. The means obtained for the intervention groups post treatment will therefore be compared to baseline values and to established normal values.

### Accelerometer activity monitoring

The accelerometer (Aipermon^® ^GmbH, Germany) will be attached to the patient's belt and positioned above the left hip. Patients will wear the device during waking hours during intervention time. The accelerometer will be attached after dressing in the morning and only taken off for showering, bathing and sleeping. In the statistical analysis a day starts at 24.00 o'clock and ends at 23.59 o'clock the same day. Mean activity per day will be calculated. All device settings (date, time, weight, age and gender) are pre-programmed for each patient upon receiving it and the device is switched on throughout the entire measurement period to keep patient handling of the accelerometer to a minimum. Upon completion of the intervention, the data is copied onto a PC, and its contents are viewed via ActiCoach MPAT2Viewer, Aipermon^®^. Wearing-time include min/day spent passively (PAS: sitting), actively (ACT: movement, but not walking), walking (WLK: 0-5 km/h)) and fast walking (FWLK: > 5 km/h). These are computed and analyzed. Walking speeds from 0 to 80 m/min are detected as *Walking *and walking speeds from 83 to 115 m/min are detected as *Fast Walking*. Speeds above 115 m/min are considered *Sportive *at which point walking would turn into jogging in most individuals. Non-wearing time is indicated by the device as "resting mode". Walking and fast walking times are added to a total walking time (TWT). Activity modes and accelerometer detection accuracy have been extensively validated [46].

### Blinding

All testing procedures will be carried out by dedicated trained, blinded assessors. A further research assistant will be responsible for data input. The data will be analysed by the main author.

### Sample size and power calculation

In order to detect a clinically significant difference in the primary outcome measure of "Timed up and go" of 10 sec (from a likely range of 10-40 sec), with a probability of 80% at a two sided 5% significance level a total of 116 patients (58 per group) must enter the study. This calculation was performed assuming a SD of the timed up and go of 19 sec [52]. Since there are indications that the distributions of the primary endpoint variable is skewed, the use of a nonparametric test should increase the actual power. To allow for drop-outs 60 participants per intervention will be recruited.

### Randomization and allocation concealment

Subjects will be randomised into cane or TheraTogs group using a computer generated randomisation programme. Allocation will be concealed with group allocation contained in centrally held sealed envelopes at Maastricht University.

### Statistical methods

The primary analysis will be performed in an intention-to-treat fashion, i.e. all subjects who where randomised and have at least a Timed Up and Go assessment before start of therapy will be included in all analyses. In the primary analysis, missing values will be replaced by the last available value. If no value under or after treatment is available, the value measured before start of therapy will be used.

The primary analysis will be performed on the Timed Up and Go after 5 weeks of therapy. A nonparametric two sample Wilcoxon-Mann-Whitney test will be performed. Significance levels will be 5%. As robustness analyses, an analysis of covariance will be performed with the value before start of therapy as covariate and centre and therapy as factors. 95% CI's will be calculated based on this analysis. Descriptive statistics for all data will be presented. For continuous data, this will include the change from pre-therapy values. Pre-therapy values will be compared between groups to identify relevant co-variables.

## Discussion - implications of the hypothesis

Various forms of walking aids are commonly used in post stroke rehabilitation although the long term effects have been sparsely researched. In order to ensure the positive effect of these treatments and to enable evidence based practice, these interventions should be clinically researched.

Early walking is an important aim of stroke rehabilitation for many reasons including the psychological well-being of the patient, to prevent loss of cardiovascular fitness or the development of secondary musculoskeletal problems. This study questions whether canes are the optimal walking aid to enable early independent walking as their use inhibits rather than stimulates activity of the hemiplegic musculature and reduces the use of automatic balance responses contrary to the aims of rehabilitation. A possible alternative to cane use was recently tested by the authors in whom the immediate effects of TheraTogs were to significantly improve hip abductor muscle activity compared to cane walking or walking without walking aids [38].

The rate of recovery of all impairments after stroke is greatest in the first few weeks and slows down after two to three months. Kwakkel et al. discussed an early window during which rehabilitation has the most long term impact [17]. The potential negative or positive effects of walking aids may therefore be particularly significant in this early stage. To our knowledge no studies have investigated this question before. The results of this study may have important clinical significance and could be used in the development of guidelines for gait rehabilitation following stroke.

## Competing interests

The authors declare that they have no competing interests.

TheraTogs orthoses have been provided by the manufacturer for use in the research, however the manufacturer has no influence on the research process, the collection and analysis of data or the presentation of results. No member of the research team is funded or benefits in any way from the manufacturer.

## Authors' contributions

CM conceived of the study, participated in its design and coordination and drafted the manuscript. JMS: participated in the design of the study and helped to draft the manuscript. FE: participated in the design and coordination of the study. BG: participated in the design of the study particularly the technical aspects of gait and movement analysis. MF: participated in the design and coordination of the study. GF: participated in the design of the study particularly statistical aspects. MJ: participated in the design and coordination of the study. AST: participated in the design and coordination of the study. RdeB: participated in the design of the study and helped to draft the manuscript. All authors read and approved the final manuscript.

## Pre-publication history

The pre-publication history for this paper can be accessed here:

http://www.biomedcentral.com/1471-2377/12/18/prepub

## Supplementary Material

Additional file 1 **Flow Chart**. Click here for file
