# Supplementary figures and images for: How to improve walking, balance and social participation following stroke: a comparison of the long term effects of two walking aids--canes and an orthosis TheraTogs--on the recovery of gait following acute stroke. A study protocol for a multi-centre, single blind, randomised control trial
Source: BMC Neurol. 2012 Mar 30;12:18. doi: 10.1186/1471-2377-12-18 (PMC3342107; doi:10.1186/1471-2377-12-18)

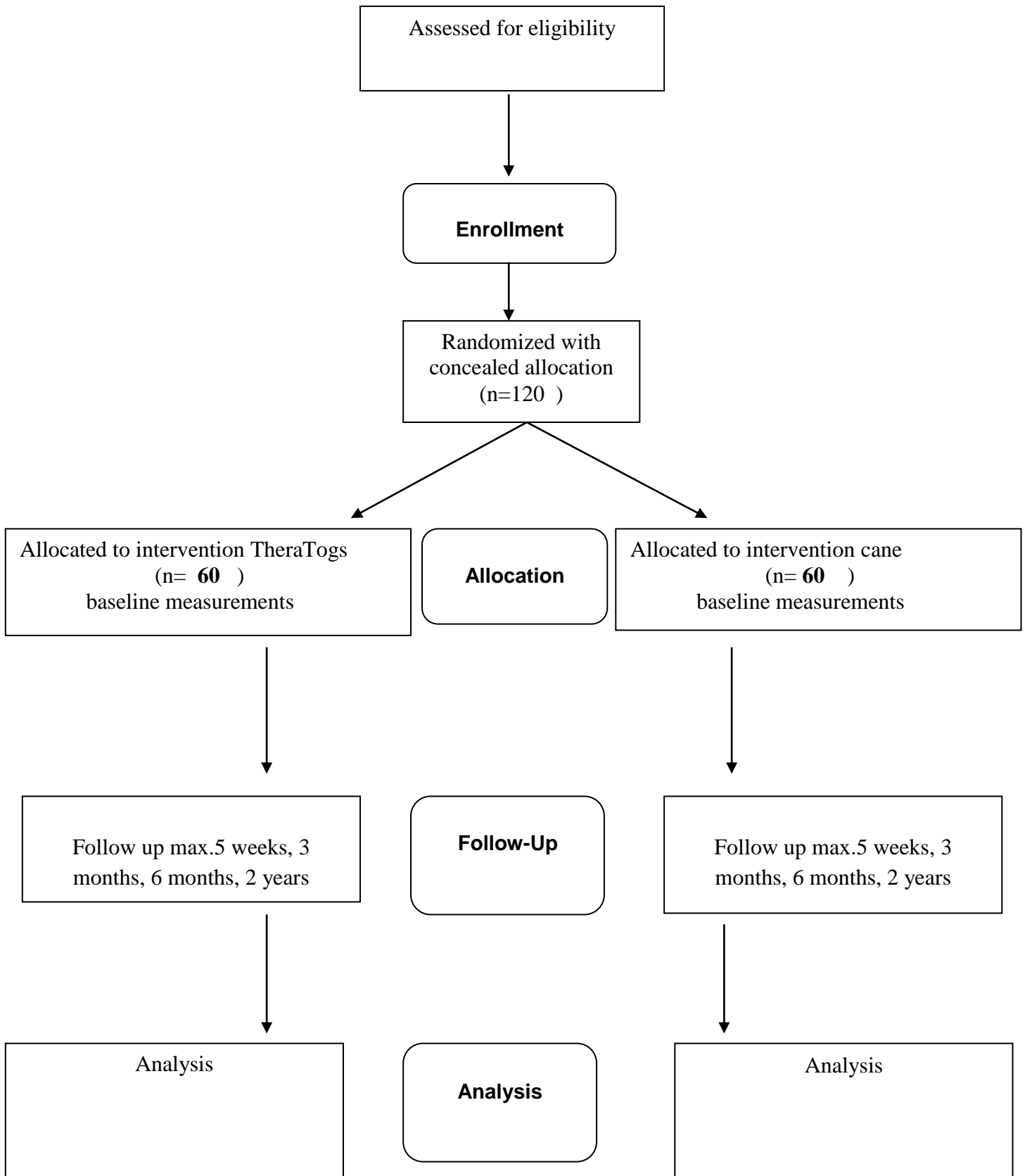

Supplement: Additional file 1 — Flow Chart. [file 1471-2377-12-18-S1.PDF]
